# FORCETRACKER: A versatile tool for standardized assessment of tissue contractile properties in 3D Heart-on-Chip platforms

**DOI:** 10.1371/journal.pone.0314985

**Published:** 2025-02-13

**Authors:** José M. Rivera-Arbeláez, Milica Dostanić, Laura M. Windt, Jeroen M. Stein, Carla Cofiño-Fabres, Tom Boonen, Maury Wiendels, Albert van den Berg, Loes I. Segerink, Christine L. Mummery, Pasqualina M. Sarro, Berend J. van Meer, Marcelo C. Ribeiro, Massimo Mastrangeli, Robert Passier

**Affiliations:** 1 MESA+Institute for Nanotechnology, BIOS Lab on a Chip Group, Technical Medical Centre, Max Planck Center for Com-plex Fluid Dynamics, University of Twente, Enschede, The Netherlands; 2 Department of Bioengineering Technologies, Applied Stem Cell Technologies Group, Technical Medical Centre, University of Twente, Enschede, The Netherlands; 3 Microelectronics, Delft University of Technology, Delft, The Netherlands; 4 Department of Anatomy and Embryology, Leiden University Medical Centre, Leiden, The Netherlands; 5 River BioMedics, Enschede, The Netherlands; Purdue University, UNITED STATES OF AMERICA

## Abstract

Engineered heart tissues (EHTs) have shown great potential in recapitulating tissue organization, functions, and cell-cell interactions of the human heart *in vitro*. Currently, multiple EHT platforms are used by both industry and academia for different applications, such as drug discovery, disease modelling, and fundamental research. The tissues’ contractile force, one of the main hallmarks of tissue function and maturation level of cardiomyocytes, can be read out from EHT platforms by optically tracking the movement of elastic pillars induced by the contractile tissues. However, existing optical tracking algorithms which focus on calculating the contractile force are customized and platform-specific, often not available to the broad research community, and thus hamper head-to-head comparison of the model output. Therefore, there is the need for robust, standardized and platform-independent software for tissues’ force assessment. To meet this need, we developed *ForceTracker*: a standalone and computationally efficient software for analyzing contractile properties of tissues in different EHT platforms. The software uses a shape-detection algorithm to single out and track the movement of pillars’ tips for the most common shapes of EHT platforms. In this way, we can obtain information about tissues’ contractile performance. *ForceTracker* is coded in Python and uses a multi-threading approach for time-efficient analysis of large data sets in multiple formats. The software efficiency to analyze circular and rectangular pillar shapes is successfully tested by analyzing different format videos from two EHT platforms, developed by different research groups. We demonstrate robust and reproducible performance of the software in the analysis of tissues over time and in various conditions. *ForceTracker*’s detection and tracking shows low sensitivity to common incidental defects, such as alteration of tissue shape or air bubbles. Detection accuracy is determined via comparison with manual measurements using the software ImageJ. We developed *ForceTracker* as a tool for standardized analysis of contractile performance in EHT platforms to facilitate research on disease modeling and drug discovery in academia and industry.

## Introduction

Cardiovascular diseases (CVD) are the main cause of death worldwide [1]. Despite all the efforts, it is still expected that the number of CVD cases will only continue to rise in the coming years. One of the bottlenecks in the development of high-efficacy treatments is inadequate *in vitro* and *in vivo* models of CVDs used in the early stage of research. To address this issue, it is required to develop *in vitro* cardiac models that recapitulate more accurately the complexity of the *in vivo* cardiac environment.

Among the most valuable approaches to improve current *in vitro* models mimicking human cardiac (patho)physiology are 3D models that use human-induced pluripotent stem cell (hiPSC) technology. These models have demonstrated their potential by having physiologically relevant cell-to-cell interactions, tissue organization and functionality [2–4]. Moreover, the use of hiPSC technology in 3D cardiac models holds promise for developing patient-specific disease models and treatments in the future.

Promising examples of 3D cardiac *in vitro* models are engineered heart tissues (EHTs) [5,6]. EHTs are composed of cardiomyocytes (CMs) and non-cardiomyocyte cells within an extracellular matrix (ECM). The cell-ECM mixture self-assembles into tissue-like constructs, supported by two or multiple anchoring points–here referred to as pillars. The pillars provide passive stretch (preload) during the tissue formation and mechanical afterload during tissue’s contraction cycles systole and diastole [7,8].

Among many benefits of using the EHT approach to model cardiac tissues *in vitro*, the one that stands out is the ease of contractile force assessment. Upon EHT formation around the pillars, tissues start contracting spontaneously in a rhythmical manner resulting in bending of the pillars. Knowing the stiffness of the pillars, the contractile force exerted by the tissues can be easily quantified by measuring the pillars’ deflection, as there is a linear relationship between the latter two.

Various EHT platforms have been developed and used across different research groups [9–11]. Many of them are based on the model of cardiac tissue suspended between two or more elastic pillars. These platforms differ in the shape and size of pillars, distance between them, as well as the shape and the size of the tissues formed around them. One aspect they all have in common is the measurement principle used to extract contractile parameters of the tissues [12–14]. Typically, deflection of the pillars is optically tracked over time and later converted into the force applied by the tissue. To assess the pillars’ deflection, different research groups use custom-made and platform-specific algorithms [15]. The adopted programming languages vary, and most of the developed tools are not publicly available to the scientific community. The mentioned drawbacks prevent cross-comparison of the results between different research groups, making the standardization of tissue contractile measurements in EHT platforms particularly challenging.

We identified the need for development of a robust, platform-independent, easy-to-use, and standalone software tool for assessing contractile properties of tissues in different EHT platforms. To address this need we propose *ForceTracker*, a standalone application developed in Python which provides a time-efficient and robust analysis of EHT contractile dynamics, from multiple video formats and for the most common shapes of anchor points in EHT platforms: circular and rectangular. The core of the standalone application is a shape-detection algorithm which tracks the deflection of tips of elastic pillars throughout all the frames of a video recording of a contractile assay. The detection is implemented in a shape-independent manner with respect to geometry of the pillars and currently operates for circular and rectangular shapes, as those are the most common shapes of pillar cross-section used in EHT platforms.

## Materials & methods

### Fabrication of EHT platforms

**Fig 1:**
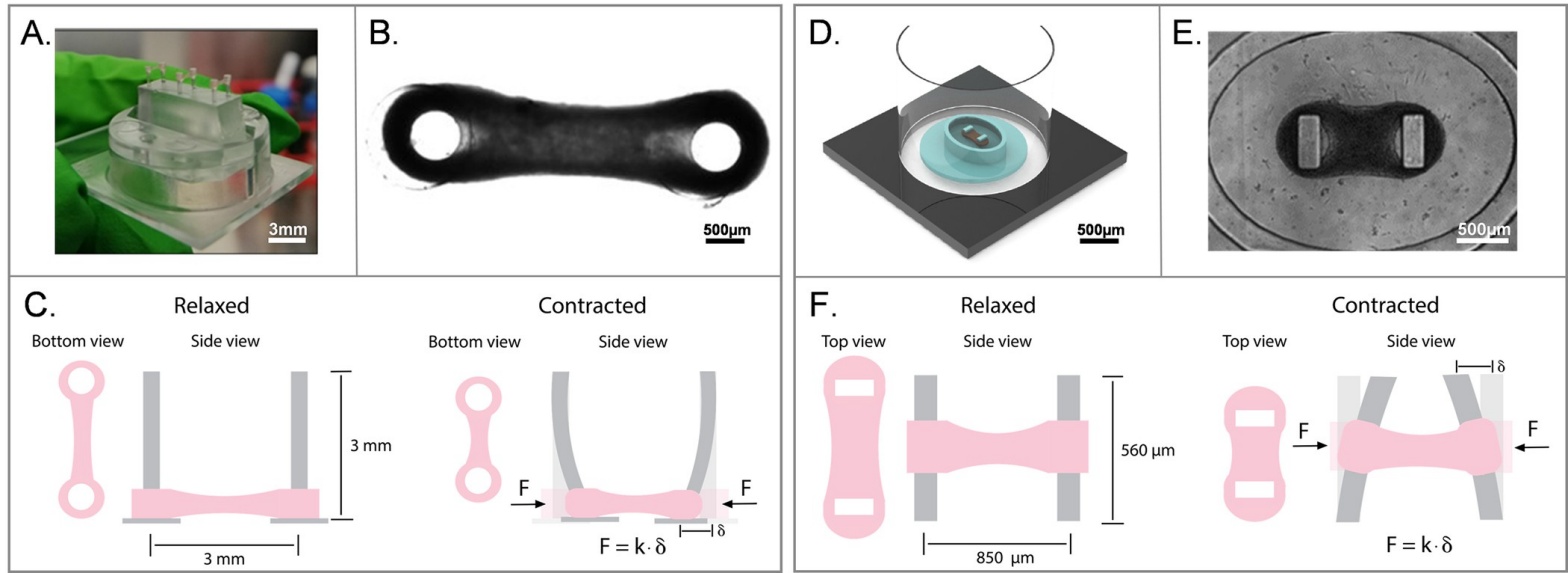
Engineered heart tissue (EHT) platforms used for the ForceTracker implementation. (A-C) Ribeiro et al.’s platform: (A) Image of three EHTs formed around cylindrical pillars on a single-well holder. (B) Brightfield image of a single EHT bottom view in a 12-well plate format. (C) Illustration of the bending principle of elastic cylindrical pillars upon tissue contraction. (D-F) Dostanic et al.’s platform: (D) 3D model of the EHT platform within a single compartment of a 96-well plate, with the PDMS structure in blue and cardiac tissue in brown. (E) Brightfield image of an EHT top view in a 96-well plate format. (F) Illustration of the bending principle of rectangular elastic pillars upon tissue contraction. F = Force of contraction, k = pillars stiffness, and *δ* = pillars deflection.

The current implementation of *ForceTracker* was benchmarked through two specific EHT platforms, as relevant representatives of the platforms used across the field (Fig 1). The first of these EHT platforms was developed at the University of Twente by Ribeiro *et al*. [7]. This platform hosts tissues suspended between two cylindrical polydimethylsiloxane (PDMS) pillars, which fit into individual holders made by micro-milling polymethyl methacrylate (PMMA) (Fig 1A). The platform is designed to fit in a 12-well plate format, with three tissues per well (Fig 1B and 1C). The second EHT platform was developed at Delft University of Technology by Dostanic *et al*. [16]. This platform consists of two rectangular micropillars within an elliptic microwell (Fig 1D), created using a combination of standard microfabrication techniques and polymer molding. The platform is made of PDMS and bonded to the bottom of a 96-well plate for high throughput analysis, as described previously [16] (Fig 1E and 1F).

### Formation of EHTs using hiPSCs

The validation of the developed software was conducted by means of the mentioned EHT platforms using the same hiPSC line (LUMC0020iCTRL-06) [17]. The hiPSCs were differentiated into CMs as described previously [7,16,18]. Briefly, in Ribeiro *et al*.’s platform [7], three tissues were formed per well in a 12-well plate format, using hiPSC-CMs and 3% of human adult cardiac fibroblasts (HCFs) from Promocell (C-12375). Cells at a final concentration of 16.8 x10^6^ cells/mL were mixed with an ECM mixture consisting of 2X Maturation medium [7], fibrinogen (final concentration 2 mg/mL, Sigma-Aldrich F8630), Matrigel (final concentration 1 mg/mL), aprotinin (final concentration 2.5 μg/mL, Sigma- Aldrich, A1153) and 0.6 U/mL of thrombin (Sigma, T7513). The EHTs for the Dostanic *et al*.’s platform [16] were composed of 70% hiPSC-CMs, 15% hiPSC-derived cardiac fibroblasts and 15% hiPSC-derived endothelial cells [18,19]. For the ECM gel mixture, 41% of acid solubilized collagen I (3.3 mg/mL), 5% of DMEM (10X), 6% of NaOH, 9% of Matrigel (final concentration 1 mg/mL) and 39% of formation media, as previously described in [20], were used. The number of cells used per tissue was approximately 31 x 10^3^ (in 2 μL volume).

### Imaging of the EHTs

Brightfield videos of both platforms were taken at 37°C in an atmosphere of 5% CO_2_, using an inverted microscope (Nikon Eclipse Ti2) with a high-speed camera, respectively Prime BSI express from Photometrics at the University of Twente and DCC3260C - High-Resolution from ThorLabs at the Leiden University Medical Center (LUMC). Contractile measurements were performed every 2 days from day 4 to day 14 after tissue formation at the University of Twente for the Ribeiro *et al*.’s platform and at LUMC for the Dostanic *et al*.’s platform. Videos were recorded for 5–10 s with a frame rate of 100 frames per second (fps) and a bit depth of 11-bit. In both platforms tissues were electrically stimulated at 2 Hz with bipolar rectangular pulses of 10 ms, 3–5 V/cm, using a custom-made pacing device connected to a pair of electrodes immersed into the well with tissues.

## Results

### *ForceTracker* implementation

#### Shape-detection and tracking algorithm

**Fig 2:**
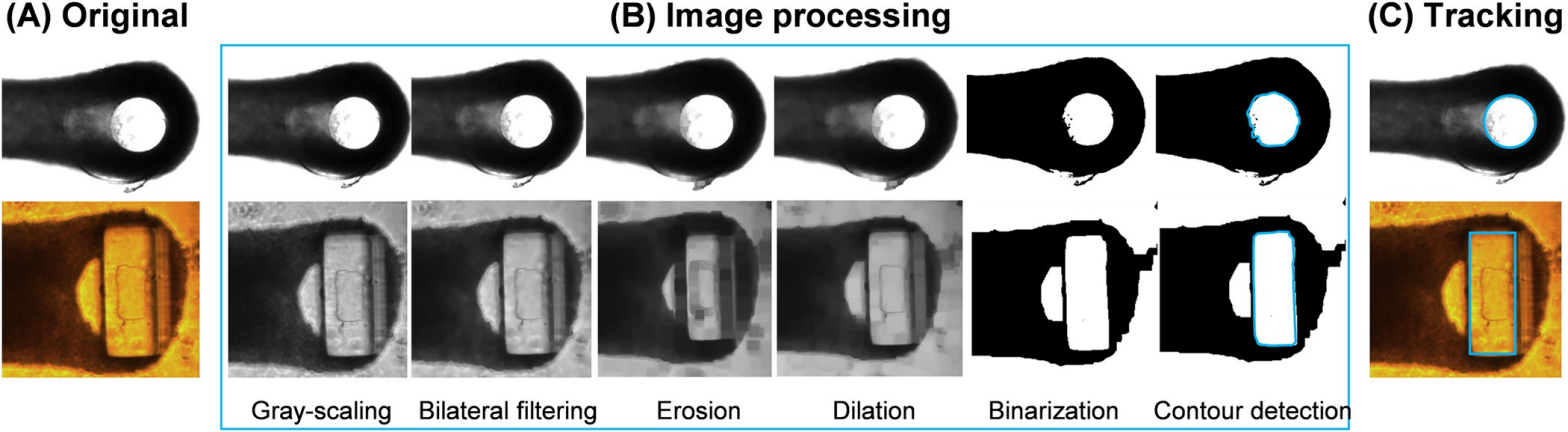
Algorithm detection steps. (A) Original image of Ribeiro et al’s (top) and Dostanic et al.’s platforms (bottom). (B) Image processing. The images are converted to grayscale, processed by the bilateral filter and lastly by complementary morphological transformations (erosion and dilation). Then, thresholding step of combining binarization and contour detection. (C) Tracking of the pillars from both platforms (light blue contours).

The core of the developed software is a shape-detection and tracking algorithm. The algorithm extracts positions and shapes of pillars’ tips from each frame of the recorded videos and tracks their displacement throughout all the consecutive frames. In addition to pillars’ displacement, the algorithm also measures and tracks the change in size of the tissue during the contraction cycles. The displacement of pillars is measured in pixels and later converted to micrometers. Further calculations correlate this displacement to the tissue’s contractile performance. The shape-detection and tracking algorithm is coded in Python, using built-in functions of openCV and skimage libraries [21]. The shape-detection and tracking algorithm was implemented in three steps: image pre-processing, thresholding, and shape tracking. All the steps of the algorithm are depicted in Fig 2 and detailed below.

1. Image pre-processing

Several image transformations are applied to ensure low noise and robust shape-detection. Firstly, each frame is converted to a grayscale image, decreasing the image complexity from three color channels to one, while preserving the information about pixel luminosity. The latter is quite important, as it is the concept that many downstream image processing algorithms rely on. The second step of the image pre-processing is removal of small noise and glitches in pixel intensity. Bilateral filtering is used for this purpose. Unlike standard blurring and smoothing techniques which only remove noise by blurring the entire image, bilateral filtering also preserves object edges and shapes [22]. This is made possible by considering only pixels with similar intensity value among neighboring groups of pixels.

However, bilateral filtering only removes small noise and reduces glitches, which is often not enough to avoid larger artifacts (e.g., shadows, air bubbles, or cells/ECM residues). In that case, morphological transformations are implemented to achieve higher-level filtering [23]. First, image erosion is performed to eliminate small objects from the image and clear out the defects around the edges of bigger objects. It results in the reduction in objects size in the image, as all the boundary pixels are ‘cleared out.’ To bring the eroded objects back to their initial dimensions, the complementary transformation to erosion, *i*.*e*. image dilation, is performed. Image dilation expands the objects by assigning value ‘1’ to the surroundings of remaining active pixels by following the pattern inferred from the transformation itself. The combination of these two morphological transformations removes most of the small defects from the original image. Additionally, it is very efficient in decreasing or even eliminating shadows of pillars from the images. The recommended number of erosion/dilation cycles is between 0 and 2. Higher number of transformations can result in distorted pillar shapes and decrease of detection accuracy. The effect of each step of image processing and its importance for the final detection result is demonstrated in Fig 2A and 2B.

2. Thresholding

Videos that are targeted for analysis with *ForceTracker* depict rhythmical contraction and relaxation cycles of 3D tissue-like constructs of cells, densely compacted around a pair of elastic pillars (S1 Fig). In both platforms of interest, the pillars are made of PDMS, a material transparent in the visible range of the electromagnetic spectrum. In the context of image processing, this transparency translates into high-brightness pixel areas representing the PDMS pillars, surrounded by low-brightness pixel areas of opaque tissue. This condition of imaging made the Otsu thresholding method the most suitable approach to distinguish transparent (pillars) from opaque (tissue) frame areas. The Otsu algorithm extracts the high-intensity objects from dark backgrounds by finding the optimal pixel threshold intensity value [24,25]. In our case, we binarize the image using Otsu threshold, by converting all the pixels belonging to the pillars’ tips into white areas, and all the pixels belonging to the tissue to black areas. The algorithm first finds the distribution of pixels per gray-scale intensity. An example of pixel intensity distribution for different brightness levels in videos is illustrated in S2 Fig. This distribution is affected by the contrast, pixel intensity and amount of bright and dark areas in an image. Regardless, in the pixel intensity distribution of each frame it is possible to clearly distinguish two peaks belonging to two classes of pixels. Low-intensity pixels in the first peak belong to the tissue area, while the high-intensity pixels of the second peak represent transparent pillars and the bright part of the image outside of tissue area. The Otsu threshold is then calculated by maximizing the variance between the two classes of pixels and finding the pixel intensity value for which the overlapping of two peaks is negligible. This output value is further used for binary thresholding the image into the white areas corresponding to the pillars and black area representing the tissue. Examples of final frames, after performing thresholding, are shown in Fig 2B.

3. Shape detection

Shape-detection and tracking start by dividing the frame vertically into two symmetric parts, using the information of the frame’s width. Each part of the image contains one pillar and approximately half of the tissue. However, precise alignment of the tissue to the center of the recording is not required for accurate detection. After the Otsu threshold is obtained, the image binarization is performed on both parts of the original image, resulting in two black-and-white images. The accuracy of final shape representation after binarization is strongly dependent on the quality of previously performed filtering and thresholding. Once the white pillar tips areas are extracted from the black background, detection can be performed. Using a contour detection function, the edges of each object in the image can be found by calculating the maximum intensity difference between neighboring pixels [26,27]. Ideally, at this step only the tissue and two pillars should be detected, as all the other artifacts should be filtered out of the frame. Exact areas of pillars are differentiated from the tissue contour with a dimension sorting algorithm. In this method the detection of pillar contour is shape-independent, as the contours can be found for any shape in the image. In this case, only validated for circular and rectangular. Depending on the shape of the pillars’ cross-section, the detected contours are approximated with circles or rectangles. Approximation with rectangles or circles is implemented only for visualization purposes and correction of the object defects (Fig 2C). Therefore, adjustment to any other pillar geometry can be implemented by simply modifying the last steps of detection.

It is relevant to mention that in the case of the platform with rectangular pillar cross-section, an additional shape often appears in the videos, between the tissue and pillars, previously described as “V-shape” [28]. This shape originates from the area without cells, as the tissue does not adhere completely to the pillars from the inner side. Due to its transparency and therefore high-intensity pixels, this area becomes white and gets merged to the one side of the rectangle. Additional masking steps are implemented to exclude the area with no cells from the final shape detection and to ensure that only rectangles are selected for tracking (S7 Fig).

For each detected pillar, shape centroid is found using image moments. In this way, the geometric center of both pillars is obtained, and its relative movement can be tracked throughout all the frames of a video. Absolute displacement of pillars relative to each other is found by calculating the Euclidian distance between the centers of the detected shapes. This calculation is repeated for every frame of a video, finally resulting in the periodic data series depicting rhythmical contraction of the tissue. The displacement is measured in pixels and later converted to micrometers for further force calculations. In addition to the pillars’ tips displacement, the change in tissue surface area is also measured in each frame. In this way, we are able to quantify the change in tissue surface area during the tissue’s contraction cycles.

#### Data analysis

The contraction wave of the tissues is the output of the shape detection algorithm, and it is used to further calculate the physiological hallmarks of the EHTs. Absolute force of contraction (FoC) is the main readout of the software, as it can be directly correlated to the performance of the CMs and their level of maturation [29]. Furthermore, FoC output allows identifying the effect of different drug compounds on the CMs, a disease mechanism, or the response to external stimuli, which can be related to the *in vivo* situation [2,29,30].

To calculate the FoC, the mechanical properties of each platform must be known (e.g., Young’s modulus of the pillars’ material, position of the tissue adherence along the pillars’ height, and geometry of the pillars). For each platform, the FoC generated by the tissues was calculated using previously described methods [7,16]. In addition to the FoC generated during contraction cycles, a resting tension acts upon the pillars during tissue formation. This tension imposes a preload to the pillars, and it is calculated as the difference between the position of the unloaded and loaded pillars. Baseline of the contraction cycle was set to zero by subtracting the resting tension from the measured force values.

Even though the FoC is a major hallmark of cardiac function, contractile kinetics also provides significant insights into cardiac (patho)physiology [2,29,30]. There are examples where pathological effects can be hindered by preserved amplitude of contractile force, but clearly expressed in the alterations of contractile kinetics [31]. Also, in the intrinsic regulatory mechanisms of myocardium, such as the Force-Frequency Relationship (FFR) and the Frank-Starling mechanism (FS), contractile kinetics are up- or downregulated.

The analysis of contractile kinetics of EHTs can be performed only across a complete contraction cycle. This is implemented in *ForceTracker* in a robust way, by analyzing the data between two minima surrounding a peak value of the contraction. In the cases when the brightfield video starts or ends in the middle of a contraction cycle, this data is discarded. Further on, speed of contraction and relaxation are calculated from each contraction cycle. Additionally, the time to reach 10% and 90% of the upstroke and downstroke of the contraction cycle are also obtained.

**Fig 3:**
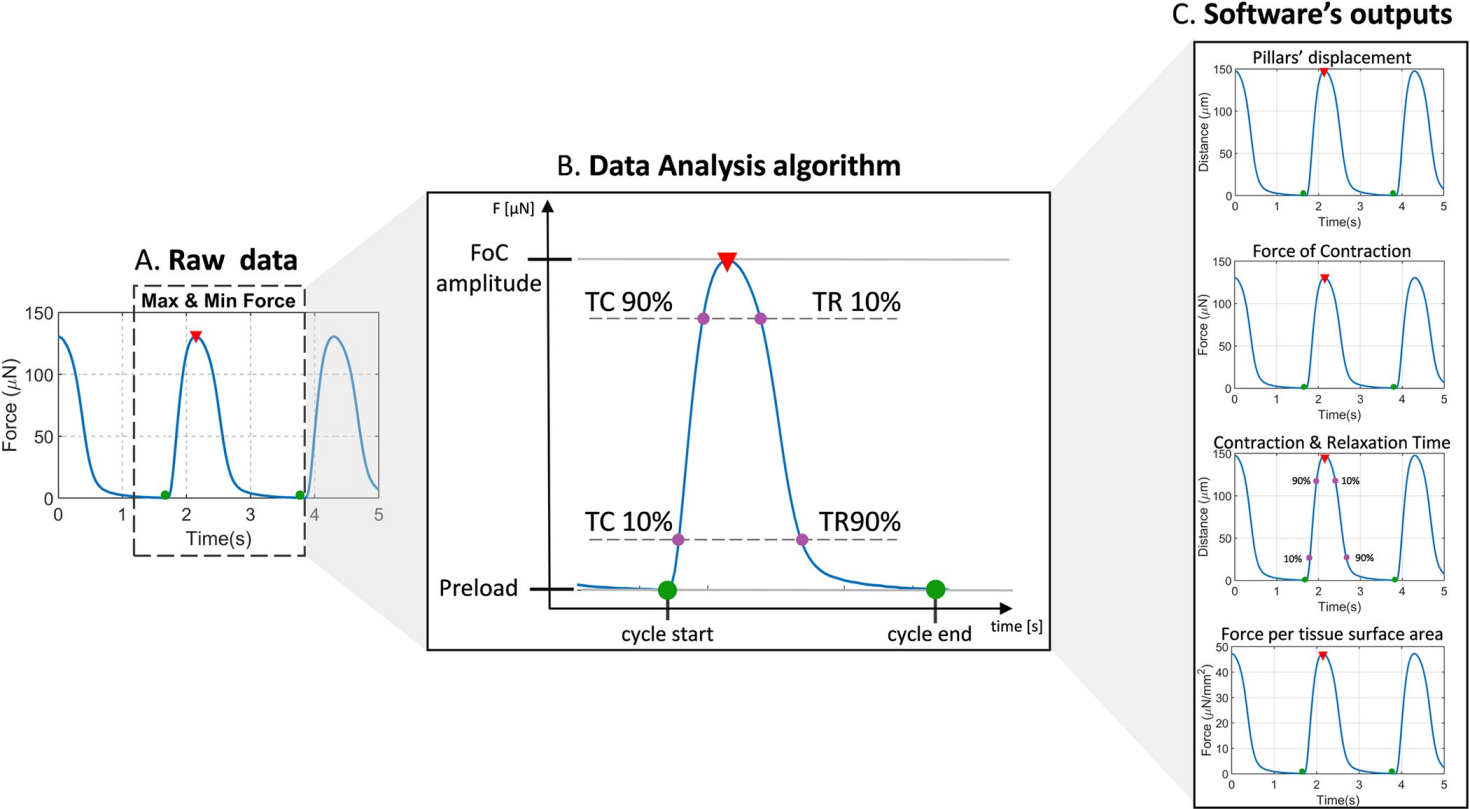
ForceTracker graphs output. (A) Force of contraction (FoC) graph with the explanation of contraction cycle with maxima and minima, and the time to achieve 10% and 90% of contraction and relaxation (B). (C) ForceTracker’s four output time series graphs: Pillars displacement, FoC, contraction and relaxation time, and force per surface area.

Another relevant contractile parameter is the FoC per cross-section area of the tissue. However, in previous studies, obtaining this value requires an additional histological step for every analyzed tissue, and can be performed only at the end of the experiment [32–37]. Instead, as an alternative information, the measured surface area of the tissue was used to calculate the force per tissue surface area. An example of the *Data analysis* output graphs and illustration of the contractile parameter calculation is shown in Fig 3.

#### Software layout

**Fig 4:**
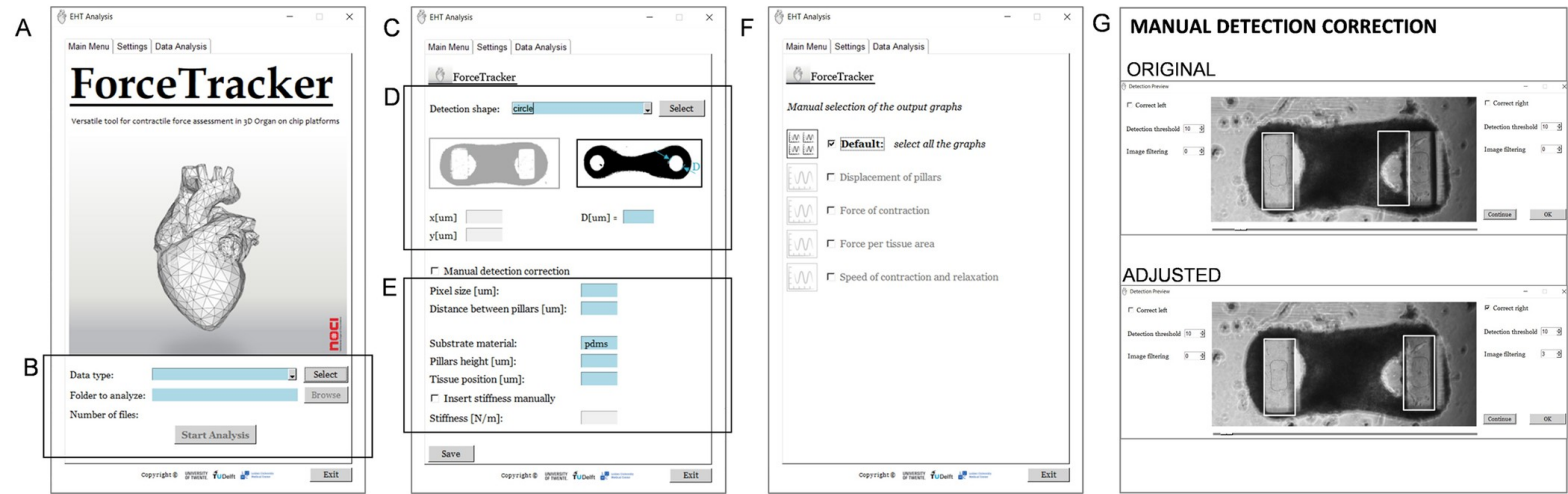
ForceTracker graphical user interface (GUI). (A) The Main window of the standalone application. (B) Section to select the type of data, the location of the files to be analyzed and the start button to start the automatic analysis. (C) Secondary window for the settings. (D) Selection of the platform and important dimensions. (E) Specifications on the material and location of the 3D tissue. (F) Data analysis window to select the output graphs of interest.

The complete *ForceTracker* app is composed of multiple building blocks. Each of the building blocks facilitates an important part of the video analysis process. The main ones are *Shape detection*, *Tracking algorithm* and *Data analysis*, which were previously detailed. However, there are additional layers required to run a robust standalone app. They are all interconnected through the user-friendly graphical interface (GUI) (Fig 4).

The software GUI was implemented using built-in functions of *PySimpleGUI* library. This library enabled us to develop an interactive environment with relevant feedback and pop-up error messages for the users [38]. The GUI contains three separate tabs requiring specific input from the user, prior to starting the analysis.

First, the algorithm requires general information such as the format of video files and the location of folder containing files for analysis. (Fig 4A) This information can be inserted via the *Main Menu tab* (Fig 4A and 4B). The second step is the definition of detection parameters within the *Settings* tab (Fig 4C). Here the user selects the cross-section of the pillars, specifies its dimensions in micrometers (Fig 4D) together with mechanical properties (Fig 4E). In the last tab *Data Analysis* (Fig 4F) the user selects the graphs stored in the output folder after completing the analysis.

**Fig 5:**
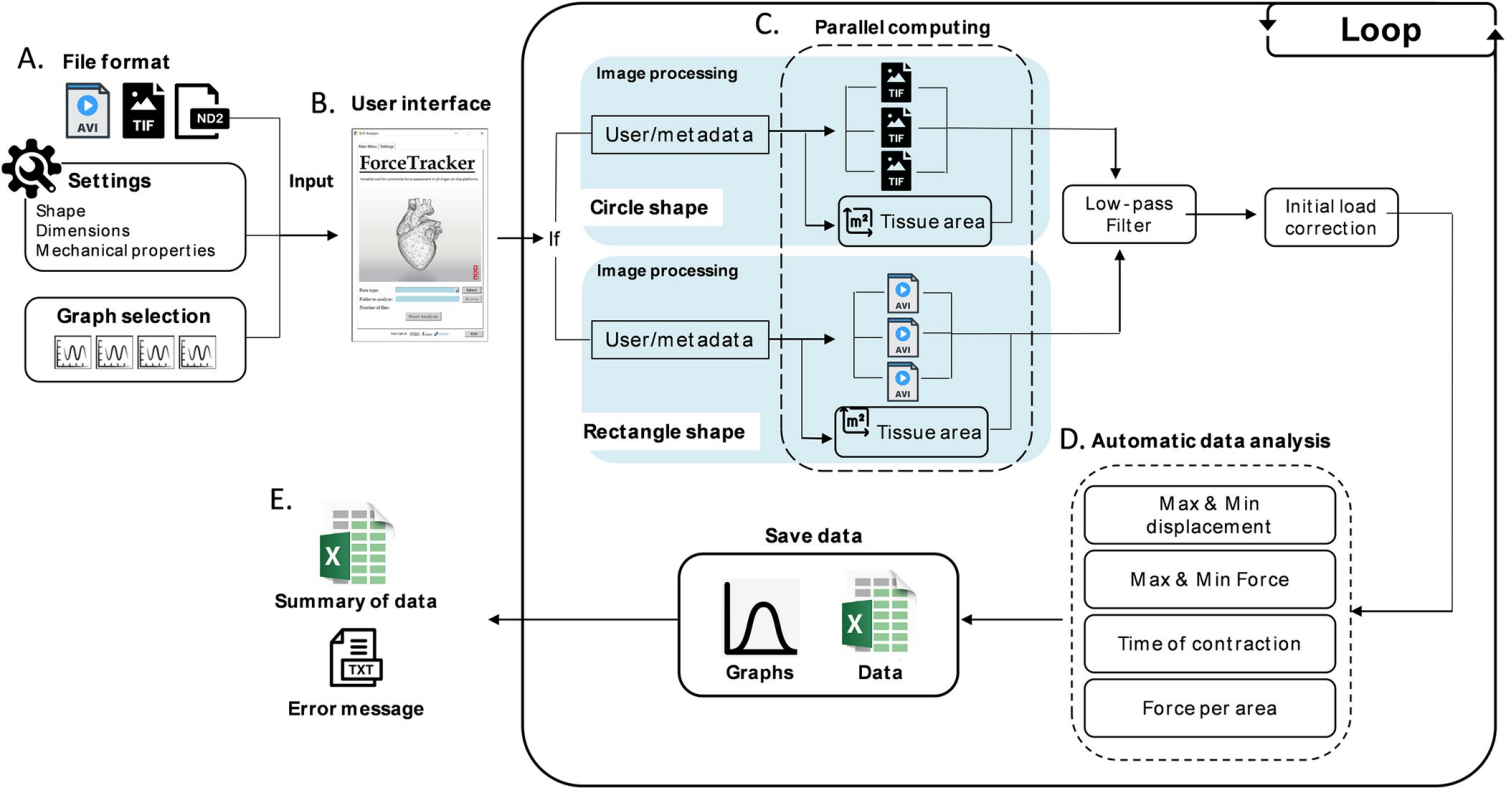
ForceTracker structure and computational flow. (A) Input files (.avi,.tif or.nd2), settings and selections of the output data. (B) Easy-to-use user interface. (C) Internal shape detection and tracking using parallel computing. (D) Automatic data analysis on the contraction wave generated by the tracking. (E) Output of the ForceTracker. Summary data and.txt log file in case of any problem with the analysis.

The automatic video analysis starts by pressing the button “Start Analysis” in the *Main Menu*, once all the input parameters are defined (Fig 5A and 5B). The computation first runs *Shape-detection and tracking algorithm* for every available video. The video analysis is done in parallel, using a multithreading approach (Fig 5C). This method maximizes the speed of data processing as it uses in parallel all available cores of a personal computer (PC) to execute the program. In our case, using a high-end PC (Intel Core i7) computation time for 50 videos (each video of approximately 10 s) without multi-thread processing was 14 min 20 s, while multithreading reduced this time to 4 min 23 s (3.3 times). During the ongoing analysis, the user is regularly updated on the remaining percentage of computation via a progress bar in the *Main Menu*. The outputs of *Shape-detection and tracking algorithm* are displacement of pillars’ tips and the change in tissue surface area for each video. The raw data are forwarded to the low-pass filter implemented as *signal*.*iirfilter* in Python. Filter parameters are set according to the data frequency, which is extracted from the raw data using Fourier transform. Filtered data become the input for the calculation of contractile parameters within the *Data Analysis* block. This computational block outputs four.*png* graphs and two excel files, one containing all the details of the video analysis for every video and the second one with the summary of all the files analyzed (Fig 5D and 5E).

The described algorithm flow applies in the case when automatic analysis is possible, *i*.*e*. if the parameters defined in the beginning of the analysis remain valid for all the videos. However, there are normally outliers and unpredictable artifacts for which manual correction of detection parameters is needed. For example, low brightness and contrast make Otsu threshold calculation challenging, as demonstrated in S2 Fig. Hence, the value of the Otsu threshold can be manually adjusted by the user to improve detection quality. Furthermore, in the case of very prominent shadows which cannot be removed during image pre-processing, it is possible to manually increase the number of dilation and erosion iterations to enhance filtering. Both options for detection improvement are available to users by choosing the *Manual detection correction* mode from the *Settings* tab. A new window appears showing detection for the currently processed video and all the parameters available for the real-time detection adjustment (Fig 4G).

Feedback is provided to the user while defining input parameters via pop-up error messages. As additional feedback, verification of the detection quality is implemented. For each video, a.*png* image showing shape detection on a single frame is stored in the main folder. In this way, fast inspection can be performed to determine the detection efficiency for all the videos.

#### Standalone application

Pursuing the goal of making a versatile tool for standardized assessment of tissues’ contractile properties in EHT platforms across different research groups, the *ForceTracker* algorithm was compiled into a standalone application. The algorithm was compiled using Nuitka [39], a Python compiler that allows compiling all the libraries and modules into a C-level program. Within this module we included *multiprocessing*, *tk-inter*, *numpy* and *pyside2* libraries. Additionally, *Mingw64* [40] was used as a C compiler. Compiling into the standalone app was executed using Python in the command prompt.

### *ForceTracker* application on two EHT platforms

To test the versatility of *ForceTracker*, brightfield videos of tissues from the two aforementioned EHT platforms (Ribeiro *et al*.[7] and Dostanic *et al*. [16]) were analyzed. More precisely, for both platforms the tissues were analyzed under different conditions to demonstrate the software capability to extract contractile parameters from a signal in the relevant frequency range. In the first case, spontaneous contractions of the tissues were recorded, as an example of low-frequency behaviour, and in the second case tissues were electrically stimulated at 2 Hz. We successfully tracked and analyzed the contractile performance of hiPSC-EHTs from both EHT platforms. Representative graphs of contraction are in S3 Fig. Each experiment was performed at least three separate times per platform.

**Fig 6:**
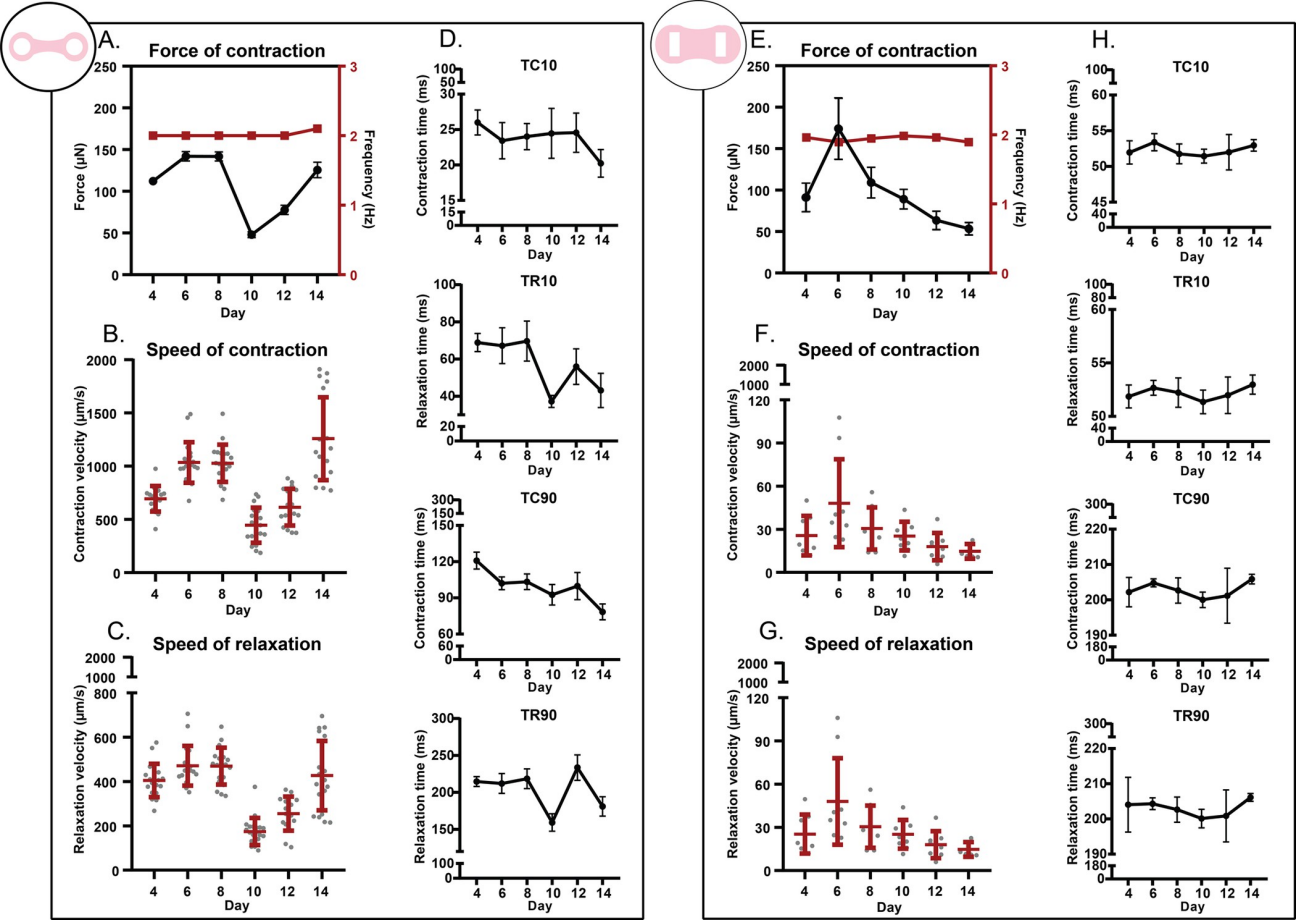
ForceTracker contractile performance analysis of two EHT platforms. (A-D) Ribeiro et al.’s platform. (A) FoC of EHTs and contraction frequency. Contraction kinetics, specifically, contraction (B) and relaxation velocity (C) of EHTs over time. (D) Time of contraction to reach 10% and 90% of the upstroke and downstroke of the contraction cycle. (E-H) Dostanic et al.’s platform. (E) FoC of EHTs and contraction frequency. Contraction kinetics, specifically, contraction (F) and relaxation velocity (G) of EHTs over time. (H) Time of contraction to reach 10% and 90% of the upstroke and downstroke of the contraction cycle. All the measurements were done at day 4, 6, 8, 10, 12, and 14. Values are expressed as means ± SEM. TC = time of contraction, and TR = time of relaxation.(N = 3, biological replicates from independent differentiations).

In the case of Ribeiro *et al*.’s platform, we observed an increase in FoC from day 4 to day 6, which then remained constant until day 8. Later, the force reached the minimum value on day 10, and increased slowly until the end of the experiment. On day 14 the value of the force was nearly as high as on day 6. All the tissues were able to follow the stimulation frequency of 2 Hz (Fig 6A). The observed change of contractile performance over the 14 days was correlated with the contraction kinetics and contraction times output (Fig 6B–6D).

The tissues formed in Dostanic *et al*.’s platform showed a steep increase in contractile performance from day 4 to day 6, when the maximum value was reached. A continuous decrease in force value followed until the endpoint of the experiment (day 14) (Fig 6E). In terms of contraction kinetics, a pattern similar to the FoC was observed, with the maximum speed of contraction and relaxation on day 6 (Fig 6F and 6G). Accordingly, on day 10 the lowest times to reach upstroke and downstroke of contraction were observed (Fig 6H).

Additionally, we analyzed the spontaneous contractions of the same tissues, prior to electrical stimulation. On average we observed that the contraction frequency of the tissues was 1.2 Hz for the tissues formed around cylindrical pillars and 0.6 Hz for the tissues on rectangular pillars. In the case of spontaneous frequency, a similar pattern in the contractile behaviour to the stimulated tissues was noticed, with a lower value of speed of contraction and relaxation (S4 Fig).

Finally, we evaluated the segmentation accuracy of *ForceTracker* by comparing the tissue surface area calculation with manually segmented tissues using the image processing software package *ImageJ*. For each time point, the first frame of the brightfield videos was used to compare the accuracy of segmentation. It is important to mention that during manual measurements the pillars’ area was subtracted from the calculated surface area, as the same principle is used by *ForceTracker*. We found that the surface area relative error of *ForceTracker* is below 5% in both platforms. Specifically, in Ribeiro *et al*.’s the accuracy is 98% on average, while for Dostanic *et al*.’s, where high level of background noise was observed, the software has an accuracy on average of 97%. Moreover, based on the surface area calculations, the relative change of tissue surface area compared to day 0 showed that during the first 5 days after tissues formation there is prominent tissue compaction in both platforms. Lastly, the force per surface area was calculated for each platform and normalized to the number of cardiomyocytes per tissue (S5 and S6 Figs).

In addition to the surface area accuracy, we also estimated the tracking accuracy of *ForceTracker*. We compared the manually measured displacement of pillars during contraction cycles in ImageJ, to the displacement detected by the software (S8 Fig). The tracking accuracy error for Ribeiro *et al*. is 6%, while for Dostanic *et al*.is 11%, on average.

## Discussion

The use of 3D *in vitro* models to mimic human physiology has been rapidly increasing in the last years. These models found application in fundamental research, drug development, disease modeling, and pharmaceutical safety. [7,9,16,41,42]. Many platforms support EHT formation and culture around rectangular or circular cross-section pillars, and mainly use image analysis to determine the contractile performance of tissues. However, there is no widely available tool that can analyze contractile performance of EHTs with different pillars’ shape, in an automatic, unbiased, reliable, and user-friendly way. Here we presented *ForceTracker*: a versatile tool for contractile performance analysis in 3D Heart-on-Chip platforms. The software addresses the need for a standardized and platform-independent measurement tool, while providing robust and computationally efficient analysis, in a user-friendly manner.

Some of the main limitations of existing software for contractile analysis are that they are platform and pillar-shape specific [9,10,43–46], which constrained their use within the scientific community. Specifically, Hansen *et al*. [10] uses software developed by a private company that tracks the top and bottom of the cylindrical pillars. This software package currently belongs to the company DiNAQOR and access to it is commercially available. In the case of Serrao *et al*. [47], a MATLAB script was developed to track specifically the center of cylindrical pillars. However, the tool automation level is low and lacks ease-of-use. This is similar to other research groups [9,29,43,48], where tailored software was designed to analyze the contractility. The output is different in each case due to computational variability in contractile measurements, as a result of non-transferable data analysis approaches. This variability in the output data makes comparison of the results from different platforms difficult and raises the question about the algorithms behind. On the other hand, MUSCLEMOTION is a software that has proven to be useful for analyzing contraction in 2D and 3D platforms, by tracking the differences in pixel intensity [49]. Nevertheless, the software outputs only a quantification of pixel intensity changes, which is correlated with displacement and thus contraction, but it cannot quantify the absolute FoC without extensive (platform-specific) calibration. This is a drawback, as the information about absolute force, initial load and tissue surface area are important for the studies of disease models, the drug compound effect or contractile performance over time [29,42,50].

*ForceTracker* fills the gap of a missing software for automated analysis of contractile performance and absolute contraction force analysis of tissues in two flexible anchoring points, with reference to the most used pillar shapes (rectangle and circle). By using a shape-independent algorithm and an easy-to-use user interface, the application has the advantage of automatically analyzing multiple videos in an unbiased way, considering the geometry and mechanical specifications of each platform. In the cases hereby studied, we used PDMS substrates due to the established fabrication protocols. However, any type of transparent or translucent anchor material that demonstrates sufficient contrast between the anchors and the tissue is suitable for the developed detection method.

The software demonstrated robustness and stability of detection over large amounts of data. Additionally, *ForceTracker* is not dependent on the symmetric position of tissues in all the frames, as each pillar is tracked independently. However, the main challenge for the detection arises from the variability of brightness and contract among videos. To circumvent this drawback, critical image pre-processing steps were successfully implemented. Nevertheless, there is still occasionally the need for manual detection correction, as the range of artifacts and variations in videos that can be anticipated by the algorithm is necessarily limited. We presently suggest to at least work with images with 738 x 266 pixels and 8-bit depth. In the future, optimal camera settings can be found and recommended to the users for video recordings.

Furthermore, *ForceTracker* facilitates the follow-up analysis with the standardized output, of the analyzed contractile properties of all the files and a detailed analysis per file, with minimal input required from the user. In this way, potential errors caused by manual interference with detection are reduced to minimum and the optimal speed of analysis is achieved. Moreover, the user has the freedom to select relevant analysis output among four available graphs (displacement, contraction force, contraction kinetics and force per surface area over time).

We also incorporated the analysis of changes in tissue surface area over time, as an additional readout of force per tissue surface area [46]. This information complements the contractile performance analysis of 3D cardiac tissues. While this readout provides information about the tissue compaction over time and during each contraction, it does not replace force per cross-section area measurements. The tissue cross-section area measurement requires an additional histological step which puts an end to the experiment, and therefore limits the throughput.

The versatility of *ForceTracker* was demonstrated by analyzing over time the contractile performance of EHTs under electrical stimulation and during spontaneous contraction. We analyzed tissues from two different EHT platforms in collaboration between three research groups (LUMC, Delft University of Technology and University of Twente). The findings we presented correspond to what was previously shown in each platform [7,16], even though a direct comparison between the platforms could not be conducted in this experiment because of differences in cell types used for tissue formation. Nevertheless, it is important to mention that by using the same hiPSC line we could observe that between day 4 and day 6 the EHTs showed the highest force of contraction in both platforms. Additionally, a significant difference in the speed of contraction was observed between the two platforms, even though the force values are comparable (Fig 6). This is related to the difference in pillars’ size and the mechanical properties of each platform. Particularly, in Ribeiro *et al*. tissue displacement during contraction cycle is larger than in Dostanic *et al* (S3 Fig). However, the effective stiffness experienced by the tissues is lower for circular-shape pillars than for rectangular ones, which explains the contractile performance. In terms of limitations, we recommend to use *ForceTracker* with videos recorded at a rate of 100 fps, and not lower than 70 fps, to capture a robust overall shape of the contraction waveforms [46–48].

## Conclusion

In this study, we proposed and validated *ForceTracker* as a versatile tool for assessment of 3D cardiac tissues cultivated in different EHT platforms using hiPSCs. By analyzing the contractile performance in two different platforms, we have shown that *ForceTracker* can be implemented and independently used across different laboratories without further software development. We demonstrated robust and stable software performance over the course of the experiments, and in various conditions. Parallel computational approach and high level of automation enabled time efficient analysis of large data sets. *ForceTracker*’s detection and tracking showed low sensitivity to common incidental defects, such as alteration of tissue shape or air bubbles. The shape detection accuracy has been verified via comparison to manual measurements using the software ImageJ.

Overall, *ForceTracker* represents a step toward standardized analysis of contractile tissue performance in 3D *in vitro* models that use flexible anchoring points. These models are not only limited to cardiac physiology. 3D tissue-like constructs are often used also on *e*.*g*. skeletal muscle platforms (Afshar et al., 2020; Iuliano et al., 2020). *ForceTracker* can be applied not only for standardized contractile measurements in EHT platforms, but also to extend the measurement principle to platforms hosting suspended tissues in two flexible anchoring points with either rectangular or circular shape, whereby generated contraction force or applied load is a required readout [51–53]. The contractile performance measurements obtained in this way provide valuable quantitative data to evaluate disease models and drug responses.

## Supporting information

S1 Fig Snapshots from EHTs videos. (A) Brightfield video of an EHT on Ribeiro *et al*.*’s* platform. (B) Brightfield video of an EHT on Dostanic *et al*.*’s* platform.
(ZIP)

S2 Fig Pixel intensity distribution. (A) Low brightness distribution. (B) Medium brightness distribution. (C) High brightness distribution.
(TIF)

S3 Fig ForceTracker representative contractile graphs. (A-D) Ribeiro et al.’s platform. (A) Tissue displacement over time. (B) FoC over time. (C) Force per surface area over time. (D) Time of contraction to reach 10% and 90% of the upstroke and downstroke of the contraction cycle. (E-H) Dostanic et al.’s platform. (E) Tissue displacement over time. (F) FoC over time. (G) Force per surface area over time. (H) Time of contraction to reach 10% and 90% of the upstroke and downstroke of the contraction cycle. Representative spontaneous contractions of the tissues shown for both platforms.
(TIF)

S4 Fig ForceTracker spontaneous contraction analysis. (A-D) Ribeiro et al.’s platform. (A) Contraction frequency. (B) FoC. Contraction kinetics, specifically, contraction (C) and relaxation velocity (D)of EHTs over time. (E-H) Dostanic et al.’s platform. (E) Contraction frequency. (F) FoC. Contraction kinetics, specifically, contraction (G) and relaxation velocity(H) of EHTs over time. All the measurements were done at day 4, 6, 8, 10, 12, and 14. Values are expressed as means ± SEM. (N = 3, biological replicates from independent differentiations).
(TIF)

S5 Fig ForceTracker tissue surface area analysis. (A-C) Ribeiro et al.’s platform. (A) ForceTracker relative error tissue surface area segmentation compared to the tissue area measured manually using Image J. (B) Relative tissue compaction compared to day 0. (C) Force per surface area over time. (D-F) Dostanic et al.’s platform. (D) ForceTracker relative error tissue surface area segmentation compared to the tissue area measured manually using Image J. (E) Relative tissue compaction compared to day 0. (F) Force per surface area over time. All the measurements were done at day 4, 6, 8, 10, 12, and 14. Values are expressed as means ± SEM. (N = 3, biological replicates from independent differentiations).
(TIF)

S6 Fig Contractile performance analysis normalized to the number of cells per tissue. (A) Ribeiro et al.’s platform. (B). Dostanic et al.’s platform. Values are expressed as means ± SEM. (N = 3, biological replicates from independent differentiations).
(TIF)

S7 Fig Steps of a masking process for "V-neck" removal. The rectangular shape is recognized for the maximum overlap of the expected top surface area of the pillar with the detected irregular shape.
(TIF)

S8 Fig Relative error of ForceTracker displacement measurement compared to ImageJ analysis. (A-B) Ribeiro et al.’s platform. (A) Normalized displacement comparison between ForceTracker and the manually-measured displacement using ImageJ. (B) Relative error of ForceTracker. (C-D) Dostanic et al.’s platform. (C) Normalized displacement comparison between ForceTracker and the manually-measured displacement using ImageJ. (D) Relative error of ForceTracker. Values are expressed as means ± SD.
(TIF)

S1 Graphical abstract (TIF)
