# Supplementary figures and images for: FORCETRACKER: A versatile tool for standardized assessment of tissue contractile properties in 3D Heart-on-Chip platforms
Source: PLoS One. 2025 Feb 13;20(2):e0314985. doi: 10.1371/journal.pone.0314985 (PMC11825004; doi:10.1371/journal.pone.0314985)

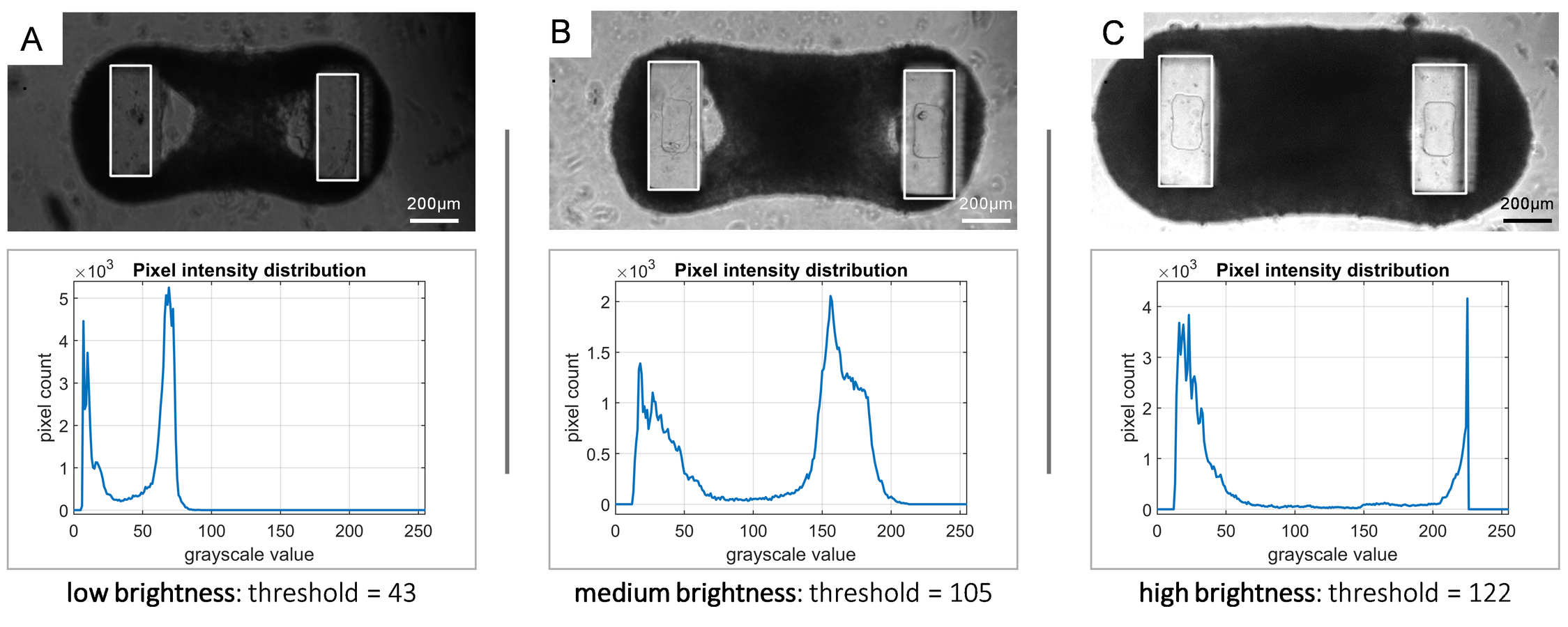

Supplement: S2 Fig — (A) Low brightness distribution. (B) Medium brightness distribution. (C) High brightness distribution. (TIF) [file pone.0314985.s002.tif]

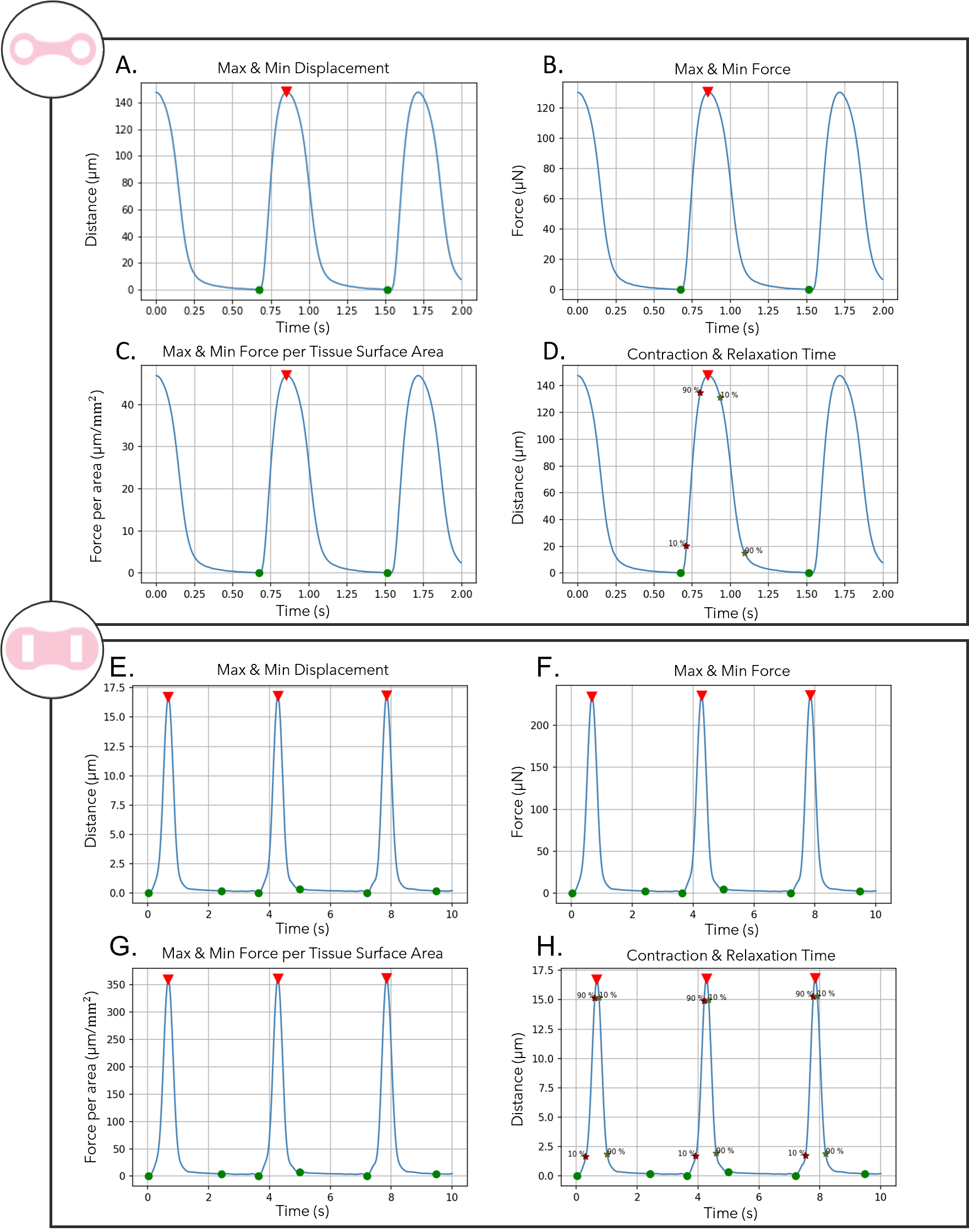

Supplement: S3 Fig — (A-D) Ribeiro et al.’s platform. (A) Tissue displacement over time. (B) FoC over time. (C) Force per surface area over time. (D) Time of contraction to reach 10% and 90% of the upstroke and downstroke of the contraction cycle. (E-H) Dostanic et al.’s platform. (E) Tissue displacement over time. (F) FoC over time. (G) Force per surface area over time. (H) Time of contraction to reach 10% and 90% of the upstroke and downstroke of the contraction cycle. Representative spontaneous contractions of the tissues shown for both platforms. (TIF) [file pone.0314985.s003.tif]

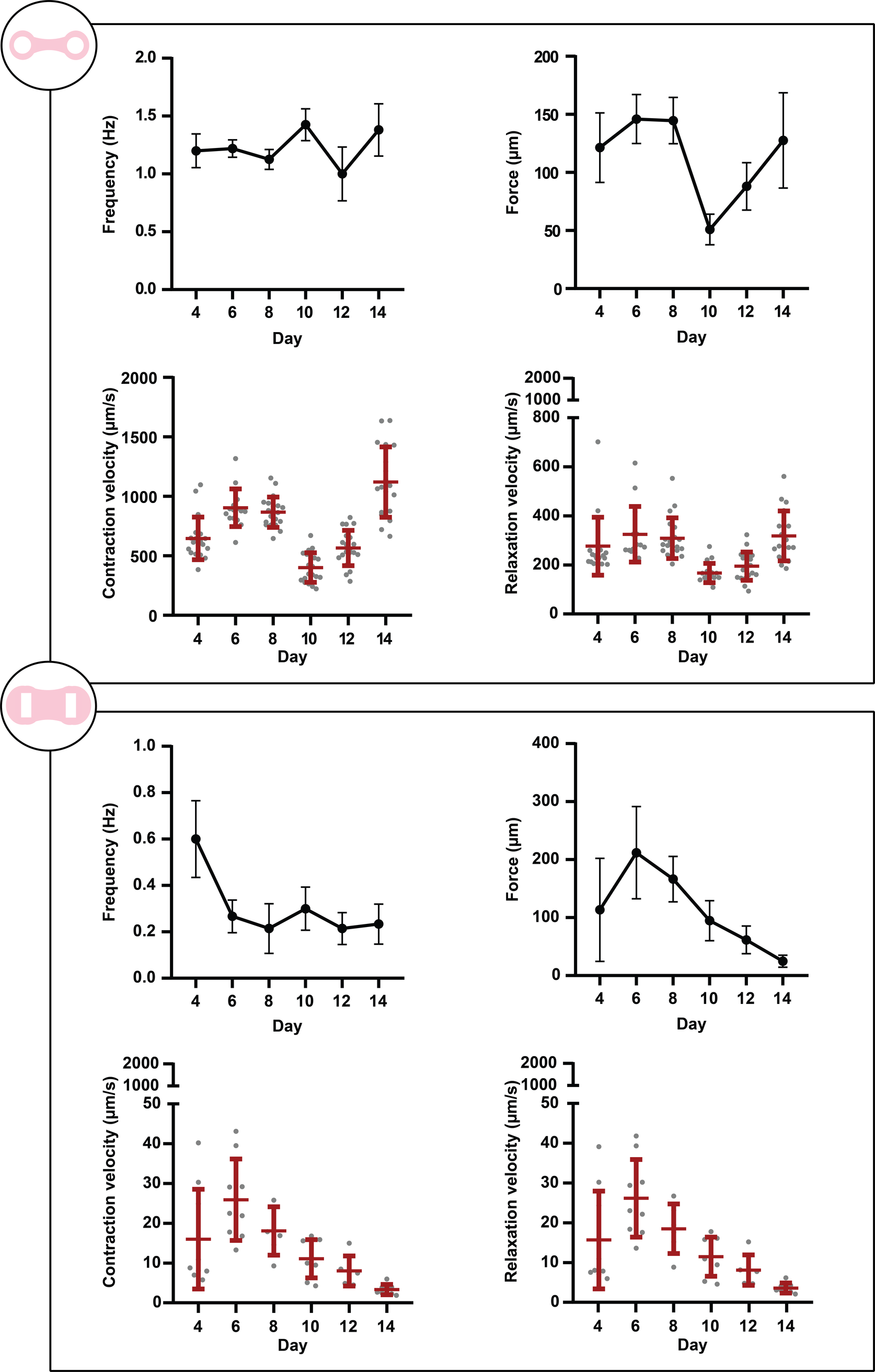

Supplement: S4 Fig — (A-D) Ribeiro et al.’s platform. (A) Contraction frequency. (B) FoC. Contraction kinetics, specifically, contraction (C) and relaxation velocity (D)of EHTs over time. (E-H) Dostanic et al.’s platform. (E) Contraction frequency. (F) FoC. Contraction kinetics, specifically, contraction (G) and relaxation velocity(H) of EHTs over time. All the measurements were done at day 4, 6, 8, 10, 12, and 14. Values are expressed as means ± SEM. (N = 3, biological replicates from independent differentiations). (TIF) [file pone.0314985.s004.tif]

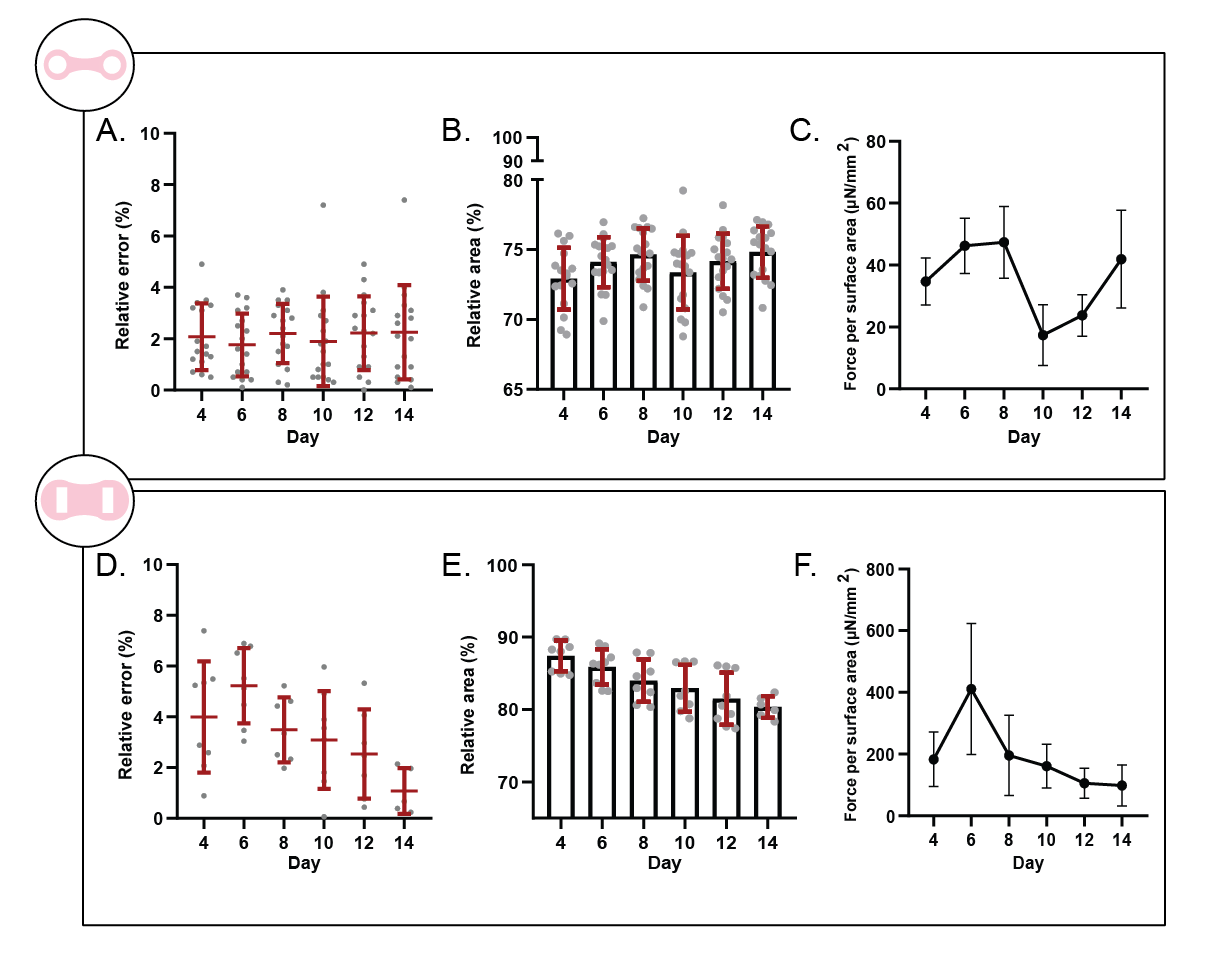

Supplement: S5 Fig — (A-C) Ribeiro et al.’s platform. (A) ForceTracker relative error tissue surface area segmentation compared to the tissue area measured manually using Image J. (B) Relative tissue compaction compared to day 0. (C) Force per surface area over time. (D-F) Dostanic et al.’s platform. (D) ForceTracker relative error tissue surface area segmentation compared to the tissue area measured manually using Image J. (E) Relative tissue compaction compared to day 0. (F) Force per surface area over time. All the measurements were done at day 4, 6, 8, 10, 12, and 14. Values are expressed as means ± SEM. (N = 3, biological replicates from independent differentiations). (TIF) [file pone.0314985.s005.tif]

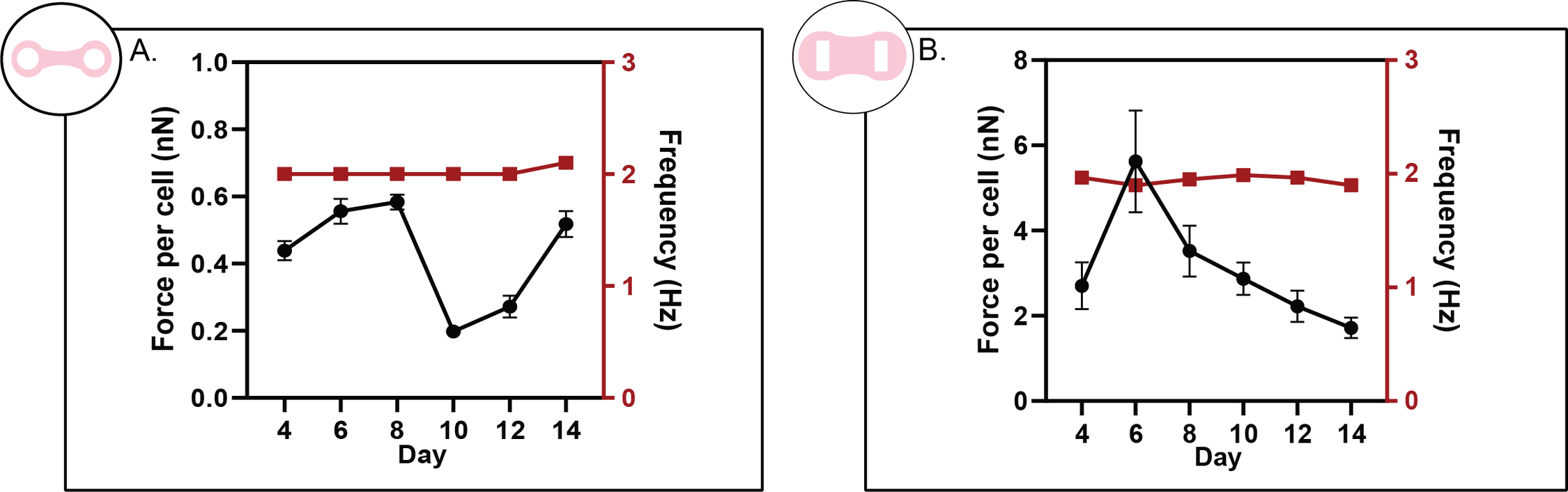

Supplement: S6 Fig — (A) Ribeiro et al.’s platform. (B). Dostanic et al.’s platform. Values are expressed as means ± SEM. (N = 3, biological replicates from independent differentiations). (TIF) [file pone.0314985.s006.tif]

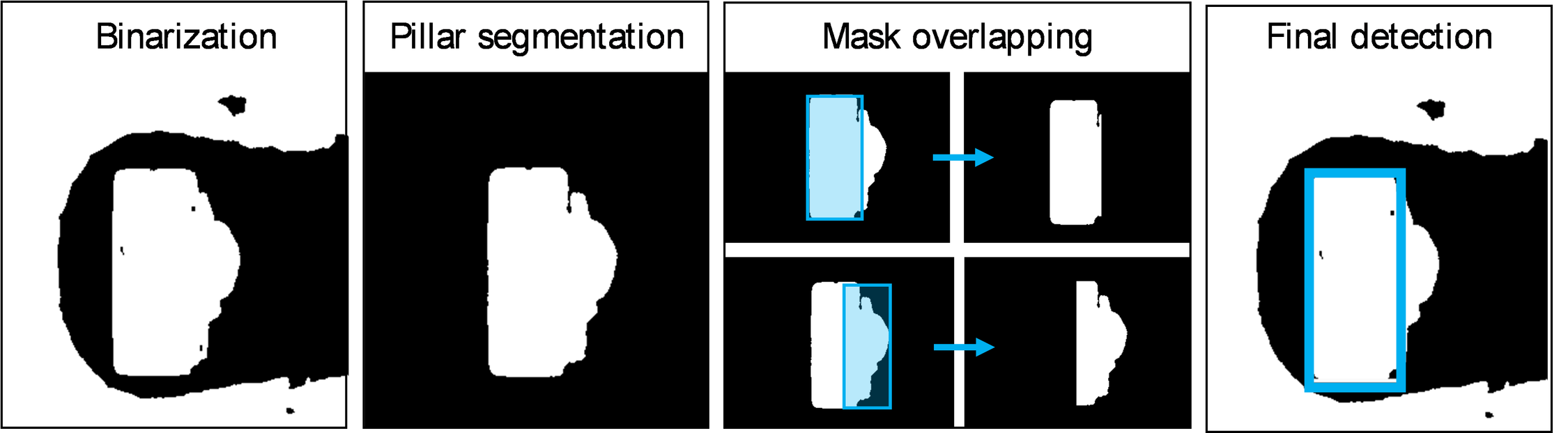

Supplement: S7 Fig — The rectangular shape is recognized for the maximum overlap of the expected top surface area of the pillar with the detected irregular shape. (TIF) [file pone.0314985.s007.tif]

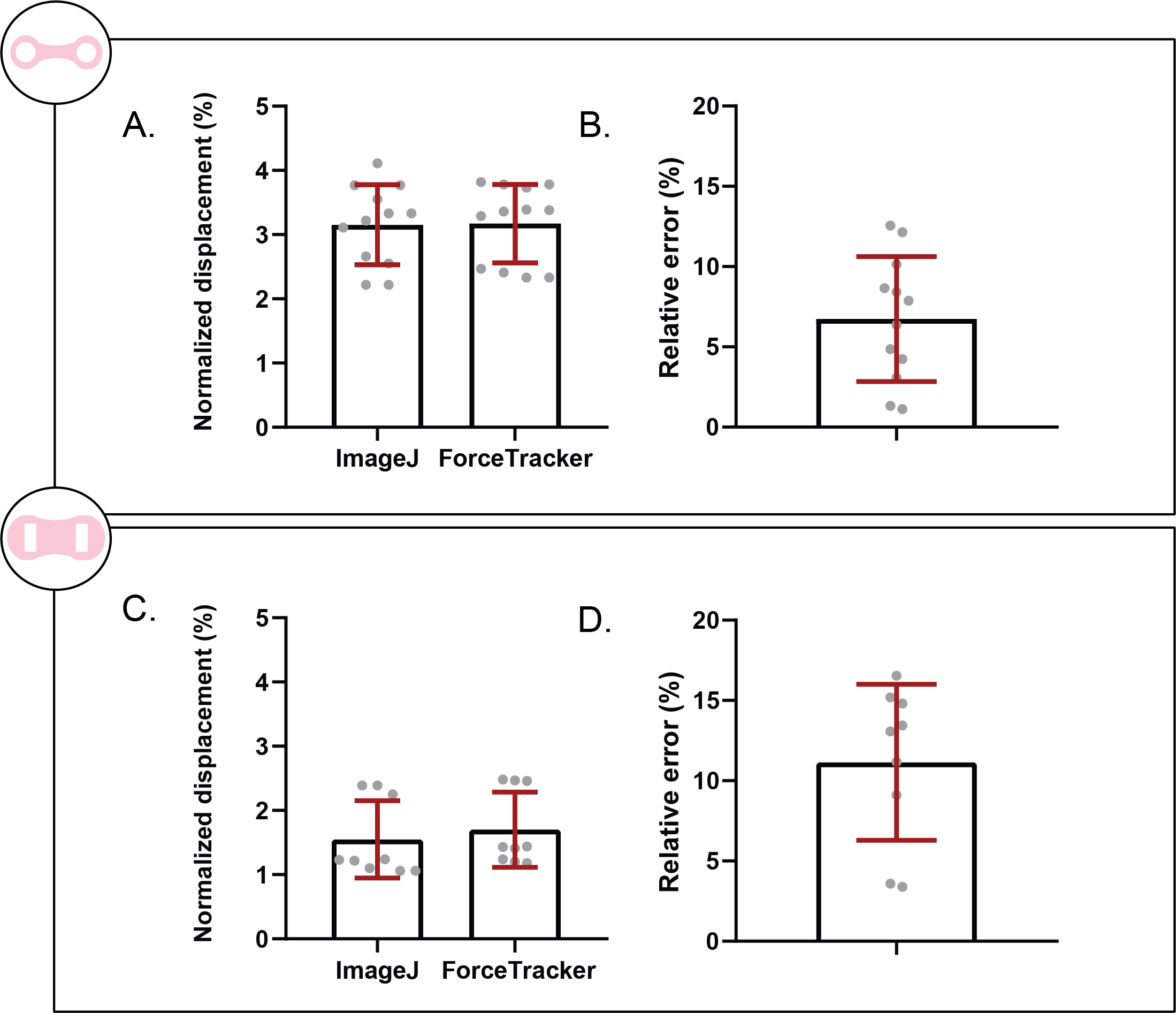

Supplement: S8 Fig — (A-B) Ribeiro et al.’s platform. (A) Normalized displacement comparison between ForceTracker and the manually-measured displacement using ImageJ. (B) Relative error of ForceTracker. (C-D) Dostanic et al.’s platform. (C) Normalized displacement comparison between ForceTracker and the manually-measured displacement using ImageJ. (D) Relative error of ForceTracker. Values are expressed as means ± SD. (TIF) [file pone.0314985.s008.tif]

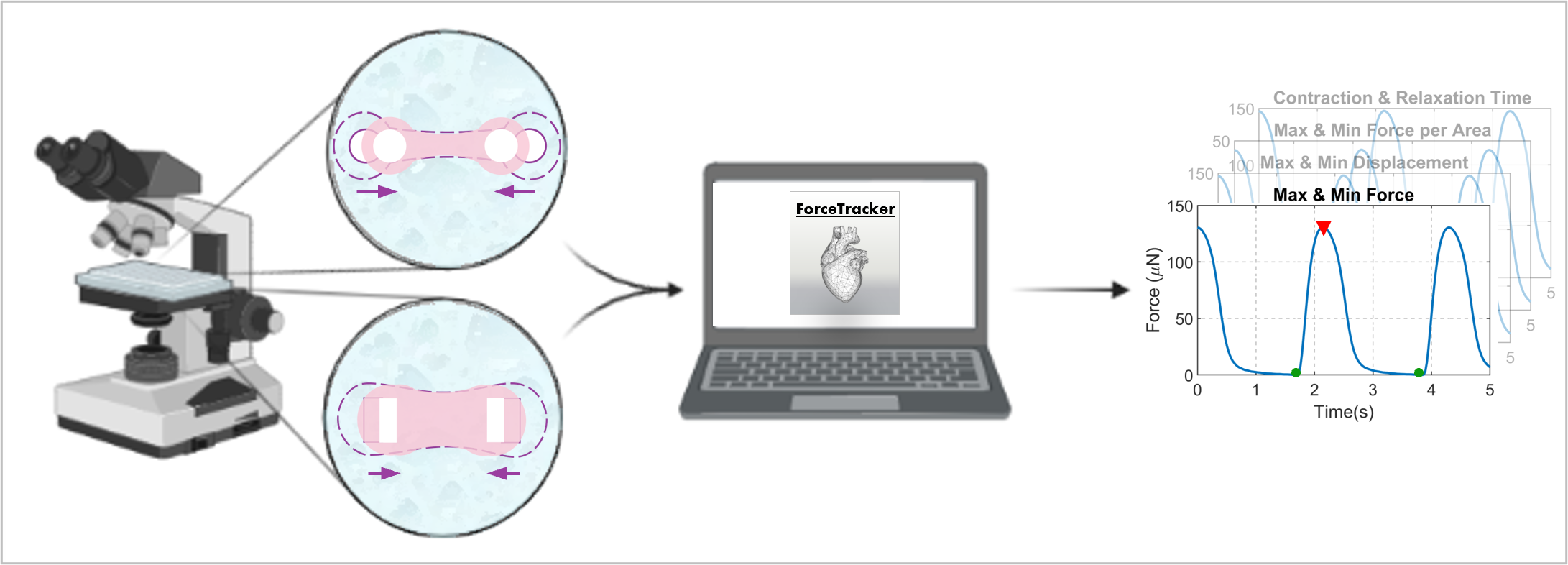

Supplement: S1 Graphical abstract — (TIF) [file pone.0314985.s009.tif]
